# Is there a lack of a glucose monitoring and management protocol for preventing hyperglycemia and glucocorticoid-induced diabetes mellitus in leprosy reactions?

**DOI:** 10.1371/journal.pntd.0012298

**Published:** 2024-07-25

**Authors:** Douglas Eulálio Antunes, Diogo Fernandes dos Santos, Jaqueline Andreoli Thomazelli, Cassio Martins Leite, Isabela Maria Bernardes Goulart

**Affiliations:** 1 National Reference Center for Sanitary Dermatology and Leprosy, Clinics Hospital, Federal University of Uberlândia, Uberlândia, Minas Gerais, Brazil; 2 State Health Department of the Federal District, Federal District, Brasília, Brazil; 3 Postgraduate Program in Health Sciences, School of Medicine, Federal University of Uberlandia, Uberlandia, Minas Gerais, Brazil; KU Leuven, BELGIUM

## Introduction

Leprosy, or Hansen’s disease, brings challenges with type 1 and type 2 reactions, linked to immune responses against *Mycobacterium leprae* antigens. Type 1 reactions, with intensified Th1 response, affect 61% of patients, while type 2 reactions, involving immune complexes, occur in 12.2% [1]. Managing neural and systemic complications relies on prednisone, a potent glucocorticoid known for its anti-inflammatory properties targeting pro-inflammatory cytokines like IL-2 and TNF-α [2]. In Brazil, corticosteroid therapy for type 1 leprosy reaction typically begins with oral prednisone at 1 mg/kg/day, decreasing by about 10 mg every 15 days until reaching 20 mg/day. From there, a reduction of 5 mg every 15 days is recommended until it drops to 5 mg/day. Maintaining this dosage for 15 consecutive days is suggested before transitioning to 5 mg/day on alternate days for another 15 days. Thus, the entire treatment lasts around 150 days (or about 5 months) [3].

Exogenous glucocorticoid exposure elevates blood glucose levels in both normal individuals and those with preexisting diabetes mellitus, leading to prediabetes, glucocorticoid-induced diabetes mellitus (GIDM), and steroid-induced hyperglycemia [4]. Higher cumulative doses exceeding 5 to 20 mg/day pose a risk for hyperglycemia [5,6]. A previous study noted dose-related type 2 diabetes risks linked to glucocorticoids, especially in immune-mediated inflammatory conditions. It found a 5% increase in diabetes risk with prednisolone doses ≥25 mg, reinforcing the dose-response relationship [7]. Before diabetes onset and hyperglycemia, prediabetes, marked by impaired glucose tolerance (IGT) and/or fasting glucose, occurs [8]. The impaired fasting glucose (IFG), with values ranging from 100 to 125 mg/dL (5.6 to 6.9 mmol/L), is identified using the fasting plasma glucose (FPG) test, which serves as a crucial initial screening tool for both diabetes and prediabetes [9]. IGT is diagnosed when plasma glucose levels range from 140 to 199 mg/dL (7.8 to 11.0 mmol/L) 2 h after ingesting a 75 g oral glucose solution, as measured by the oral glucose tolerance test (OGTT). This test is more comprehensive and is commonly employed in diagnosing diabetes and gestational diabetes [9]. The definition of GIDM involves abnormal blood glucose levels in individuals without preexisting diabetes, presenting challenges in detection and potentially leading to underestimation in clinical practice [3]. The complexity in diagnosing GIDM arises from the choice of diagnostic test, with physicians frequently favoring the FPG test over the OGTT that exhibits better performance in assessing the impact of glucocorticoids on postprandial glycemia [10].

In the context of leprosy reactions, the glycated hemoglobin test (HbA1c) serves as an indicator of long-term glycemic control, routinely accompanied by a hemogram (red blood cell count) for a comprehensive assessment and precise interpretation of diabetes, particularly in patients with conditions such as anemia, which may be associated with increased HbA1c levels [9–11].

Moreover, for patients on prednisone, requesting serum lipase and amylase tests is paramount. Low levels of these enzymes in diabetes patients correlate with elevated blood glucose levels due to impaired insulin action, stemming from insulin resistance or inadequate insulin secretion. Reduced levels of amylase and lipase are associated with both type 1 and type 2 diabetes mellitus, metabolic syndrome, and excess adiposity [12].

In this context, for both diagnosing GIDM and managing leprosy reaction, capillary blood glucose monitoring is crucial. For patients without preexisting diabetes, monitoring blood glucose once daily before lunch or the evening meal, or 1 to 2 h after these meals is preferable. However, for those already diagnosed with diabetes, monitoring blood glucose up to 4 times daily is recommended—before and after meals, as well as before bedtime. This practice is advised regardless of the level of diabetes control after 2 h [5,13].

Despite the prevalent nature of leprosy and its associated reactions, a significant data void exists, particularly concerning patients undergoing corticoid therapy in endemic regions. Varied incidence rates of prediabetes and GIDM, as highlighted by existing studies, underscore the pressing need for a standardized protocol to navigate the complexities of managing patients on prolonged steroid regimens.

## Our viewpoint

Few studies have reported significant findings regarding glucocorticoids, leprosy, and metabolic disorders, and none have elaborated on a protocol assessment to manage blood glucose levels during glucocorticoid use.

One study, highlighted in the literature, estimated that approximately 37.7% of leprosy patients experienced prediabetes, with an incidence rate of 20% specifically in cases of lepromatous leprosy [14]. Additionally, a prior study observed that 23.5% of individuals with leprosy developed GIDM under varying doses of prednisone [15].

Currently, there is no research specifically addressing glucose management protocols aimed at preventing hyperglycemia and GIDM in individuals experiencing leprosy reactions. The absence of established protocols indicates a gap in comprehensive strategies for monitoring glucose levels in these patients, before, during, and after the prescription of prednisone.

According to the lack of reference literature, we are convinced that there are no glucose management protocols for monitoring leprosy reactions. However, to support our viewpoint and determine the prevalence of IFG, a factor associated with an increased risk of hyperglycemia related to steroid therapy, we analyzed the plasma fasting glucose levels of 75 live patients affected by leprosy reactions, as recorded in a secondary database. These patients were categorized into 4 groups based on the duration of prednisone usage. All patients commenced treatment for leprosy reaction with 1 mg/kg body weight (approximately 60 mg/day), gradually reducing the dosage until reaching 5 mg/day. Continuous prednisone usage was observed only in the first group, which utilized it for up to 4 months (from 60 to 10 mg/day). For those who used the corticosteroid for more than 4 months (second, third, and fourth groups), the total duration of usage represents an accumulation obtained from the sum of continuous periods interspersed with intermittent periods of prednisone usage, in accordance with the onset of new leprosy reactions. The patients selected were those whose fasting blood glucose levels were measured during the period when the corticosteroid dosage was equal to or greater than 40 mg/day. A 1-month period was subtracted from the final count of prednisone usage concerning the dose of 5 mg/day.

In our investigation, the overall prevalence of IFG was 10.6% (8/75). Although Brazilian clinical protocols recommend administering prednisone at a dosage of 1 mg/kg regardless of age, our study identified 13 patients aged 70 years or older who followed a personalized treatment regimen, initiating therapy with 40 mg/day of prednisone. S1 Table shows the epidemiological and clinical characteristics categorized by cumulative prednisone usage groups.

**Fig 1:**
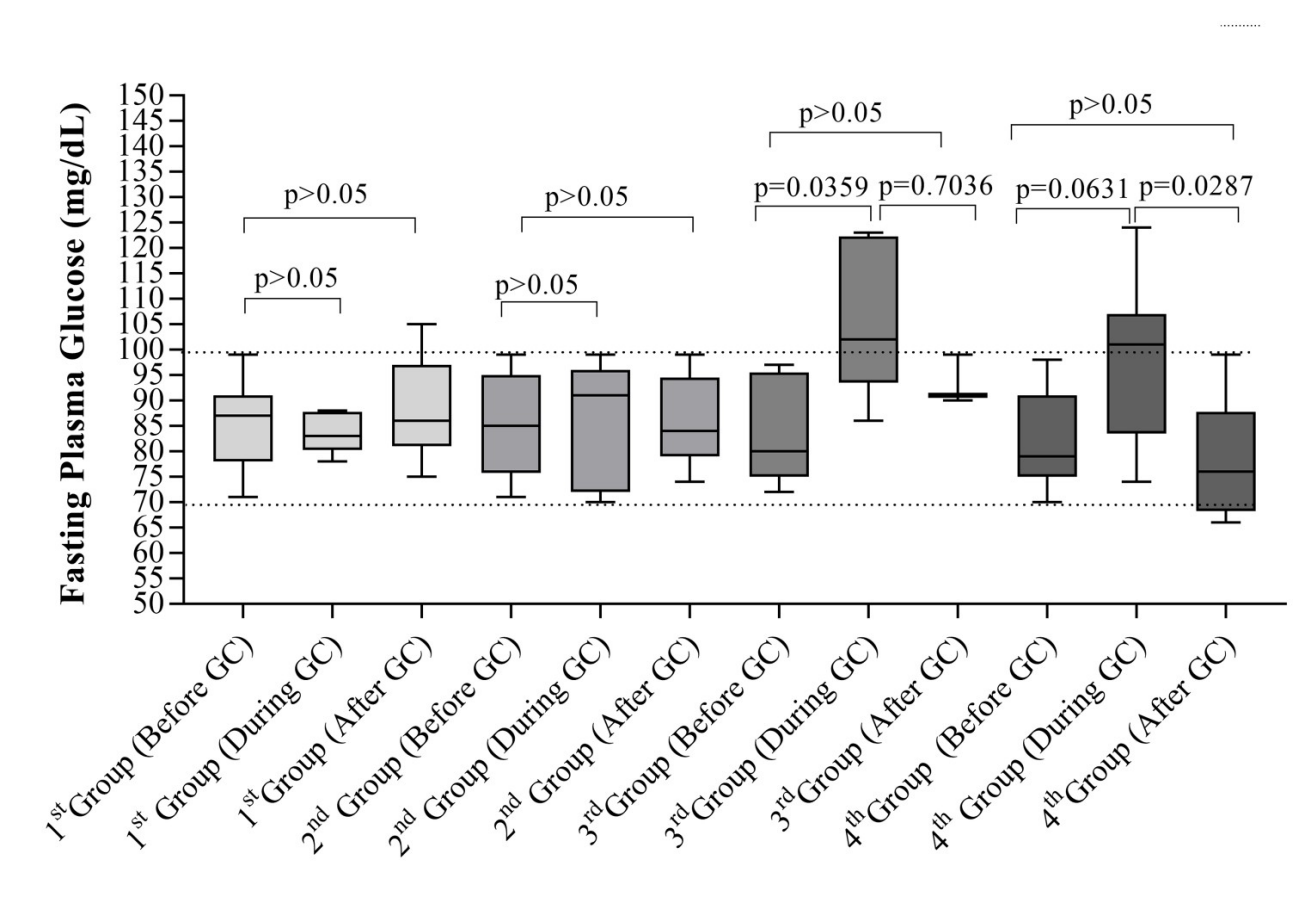
Fasting plasma glucose levels of leprosy reaction groups classified by cumulative prednisone dose and sample collection timing (Before, During, and after Glucocorticoid Usage)/Abbreviations/notes: GC—Glucocorticoid (Prednisone), Lower-Level Control (70 mg/dL), Upper-Level Control (100 mg/dL).

Fig 1 displays the non-paired Kruskal–Wallis test, comparing FPG levels before, during, and after the use of prednisone among different groups, as per the previously cited methodology. The box plot descriptive analysis reveals a notable increase in median glucose levels during prednisone use in the third (median: 102 mg/dL; Min.: 86 mg/dL/Max.: 123 mg/dL) and fourth groups (median: 101 mg/dL; Min.: 74 mg/dL/Max.: 124 mg/dL), both of which had a cumulative prednisone dose exceeding 8 months (equivalent to more than 2 leprosy reactions). These findings draw our attention since both groups were under prednisone treatment during blood sample collection, comprised older patients experiencing more than 2 reactional states, and consequently exhibited IFG. It underscores the necessity to implement a clinical protocol for monitoring these patients and managing their glucose levels through comprehensive tests to prevent GIDM and hyperglycemia.

All patients underwent detailed assessments of hepatic, renal, and hematological functions, along with a meticulous review of medical records to exclude those with chronic non-communicable diseases and other infectious conditions. Patients with diabetes were also rigorously excluded from the analysis.

**Fig 2:**
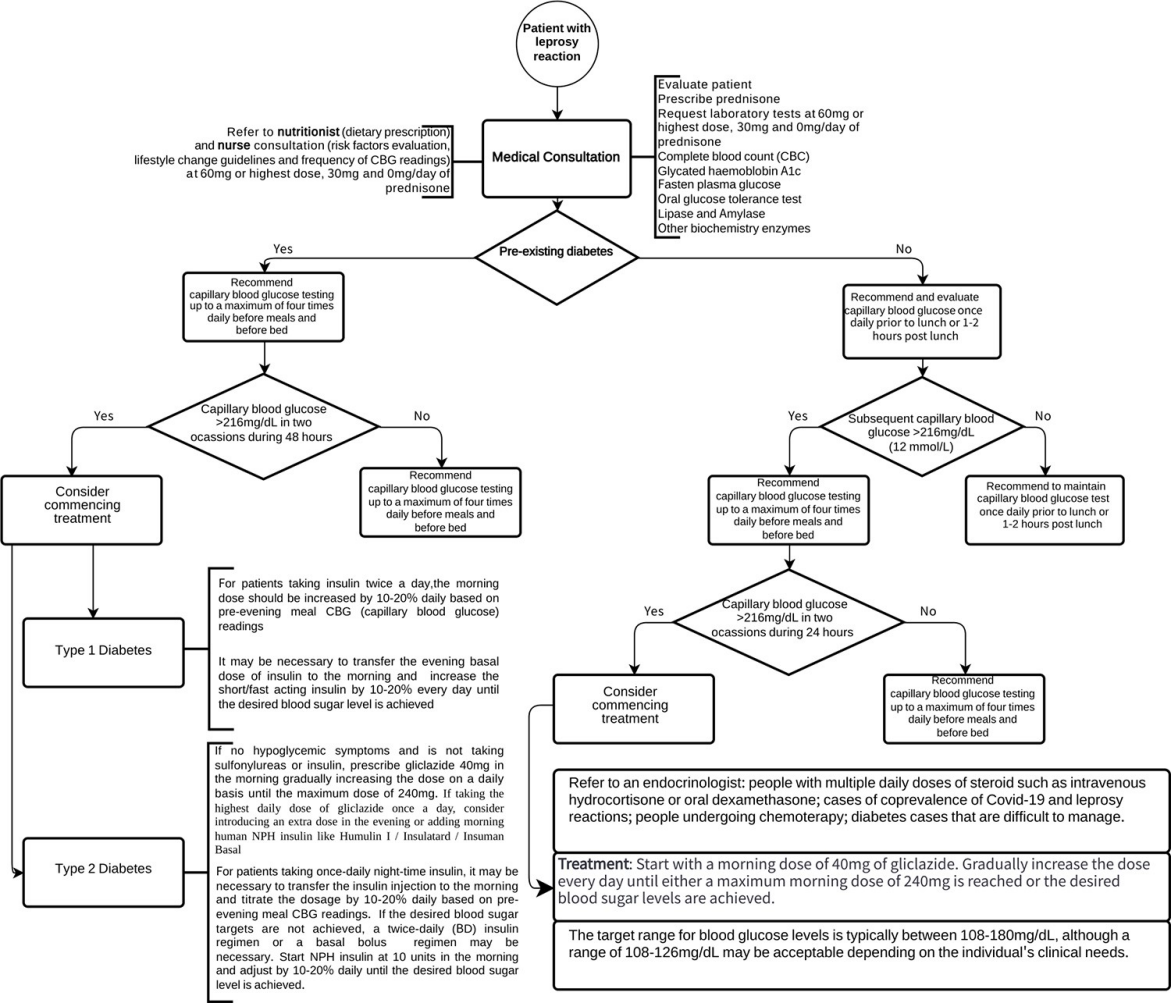
The suggested glucose monitoring and management protocol for assistance of leprosy reactions patients who are taking corticoids based on the guideline from the JBDS.

Considering these results, we propose adopting this glucose monitoring and management protocol (Fig 2) for guiding professional decisions and assisting patients undergoing corticoid treatment during leprosy reaction events, based on the Joint British Diabetes Societies (JBDS) guideline [5]. This protocol aids healthcare providers in preventing hyperglycemia and GIDM in outpatients/inpatients on prolonged steroid therapy.

We believe that, despite some studies addressing the prevalence of metabolic disorders associated with leprosy, there is an undisclosed prevalence among these patients, especially in endemic countries, due to the absence of blood glucose tests before, during 30, and 60 mg of prednisone and after the use of this medication. We recommend medical consultation with requests for FPG, OGTT, glycated hemoglobin, complete blood count, lipase, and amylase tests before initiating prednisone. After 30 and 60 mg of prednisone, we advise all laboratory tests except for lipase and amylase. We suggest consultations with nutritionists and nurses, following the recommended protocol. In specific cases, physicians should refer patients to endocrinologists. We propose, in alignment with JBDS, capillary blood glucose tests before meals and bedtime, with the frequency adjusted based on preexisting diabetes. Treatment for diabetes cases (type 1 or 2) is fundamental according to the glucose monitoring outlined in the suggested protocol.

## Conclusions

We strongly advocate for implementing a glucose monitoring and management protocol, emphasizing the critical importance of regular blood glucose monitoring, quoted in this viewpoint. This protocol plays a pivotal role in safeguarding leprosy reaction patients undergoing corticoid treatment, effectively preventing potential metabolic disorders associated with steroids, particularly GIDM.

## Ethical considerations

This study was approved by the Research Ethics Committee of the Federal University of Uberlândia—Brazil (CAAE: 42880720.7.0000.5152). As it involves the collection of secondary data from electronic medical records, the informed consent form was waived.

## Supporting information

S1 Table Clinical and epidemiological characteristics of leprosy reaction patients categorized by duration of prednisone use into 4 groups. Abbreviations/notes: BT—Borderline Tuberculoid; BB—Borderline Borderline; BL—Borderline Lepromatous; LL—Lepromatous Leprosy; MB: Multibacillary; PB: Paucibacillary. *—BT classified as MB based on ELISA anti-PGL-1, neural thickening, qPCR slit skin smear, and biopsy.
(DOCX)

## References

[1] Ambrosano L, Santos M, Machado E, Pegas ES. Epidemiological profile of leprosy reactions in a referral center in Campinas (SP), Brazil, 2010–2015. An Bras Dermatol. 2018;93:460–461. doi: 10.1590/abd1806-4841.20187260 29924248 PMC6001109

[2] Puckett Y, Gabbar A, Bokhari AA. Prednisone. StatPearls. Treasure Island (FL) ineligible companies. Disclosure: Aishah Gabbar declares no relevant financial relationships with ineligible companies. Disclosure: Abdullah Bokhari declares no relevant financial relationships with ineligible companies. 2024.

[3] Brasil. Ministério da Saúde. Secretaria de Vigilância em Saúde, Departamento de Doenças de Condições Crônicas e Infecções Sexualmente Transmissíveis. Protocolo Clínico e Diretrizes Terapêuticas da Hanseníase. Brasília: Ministério da Saúde; 2022. p. 152. Available from: https://bvsms.saude.gov.br/bvs/publicacoes/protocolo_clinico_diretrizes_terapeuticas_hanseniase.pdf [accessed 2024 Feb 28].

[4] Shah P, Kalra S, Yadav Y, Deka N, Lathia T, Jacob JJ, et al. Management of Glucocorticoid-Induced Hyperglycemia. Diabetes Metab Syndr Obes. 2022;15:1577–1588. doi: 10.2147/DMSO.S330253 35637859 PMC9142341

[5] Roberts A, James J, Dhatariya K. Joint British Diabetes Societies for Inpatient Care. Management of hyperglycaemia and steroid (glucocorticoid) therapy: a guideline from the Joint British Diabetes Societies (JBDS) for Inpatient Care group. Diabet Med. 2018;35:1011–1017. Available from: https://abcd.care/sites/default/files/site_uploads/JBDS_Guidelines_Current/JBDS_08_Management_of_Hyperglycaemia_and_Steroid_%28Glucocorticoid%29_Therapy_with_QR_code_January_2023.pdf [accessed 2024 Feb 27]. 30152586 10.1111/dme.13675

[6] Low Wang CC, Draznin B. Use of Nph Insulin for Glucocorticoid-Induced Hyperglycemia. Endocr Pract. 2016;22:271–273. doi: 10.4158/EP151101.CO 26789338

[7] Wu J, Mackie SL, Pujades-Rodriguez M. Glucocorticoid dose-dependent risk of type 2 diabetes in six immune-mediated inflammatory diseases: a population-based cohort analysis. BMJ Open Diabetes Res Care. 2020;8. 10.1136/bmjdrc-2020-001220 PMC7389515 32719077

[8] Lawal Y, Bello F, Kaoje YS. Prediabetes Deserves More Attention: A Review. Clin Diabetes. 2020;38:328–338. doi: 10.2337/cd19-0101 33132502 PMC7566925

[9] Khan RMM, Chua ZJY, Tan JC, Yang Y, Liao Z, Zhao Y. From Pre-Diabetes to Diabetes: Diagnosis, Treatments and Translational Research. Medicina (Kaunas). 2019;55. doi: 10.3390/medicina55090546 31470636 PMC6780236

[10] Nowak KM, Rdzanek-Pikus M, Romanowska-Prochnicka K, Nowakowska-Plaza A, Papierska L. High prevalence of steroid-induced glucose intolerance with normal fasting glycaemia during low-dose glucocorticoid therapy: an oral glucose tolerance test screening study. Rheumatology (Oxford). 2021;60:2842–2851. doi: 10.1093/rheumatology/keaa724 33254223

[11] Janice D, Prathima MB, Sushith S, Narayanan R, Reshma S, Nair S, et al. Effect of iron deficiency anaemia over glycated hemoglobin in non-diabetic women. Int J Biochem Mol Biol. 2022;13:23–27. 35891642 PMC9301143

[12] Ko J, Cho J, Petrov MS. Low serum amylase, lipase, and trypsin as biomarkers of metabolic disorders: A systematic review and meta-analysis. Diabetes Res Clin Pract. 2020;159:107974. doi: 10.1016/j.diabres.2019.107974 31811884

[13] Aberer F, Hochfellner DA, Sourij H, Mader JK. A Practical Guide for the Management of Steroid Induced Hyperglycaemia in the Hospital. J Clin Med. 2021;10. doi: 10.3390/jcm10102154 34065762 PMC8157052

[14] Saraya MA, Al-Fadhli MA, Qasem JA. Diabetic status of patients with leprosy in Kuwait. J Infect Public Health. 2012;5:360–365. doi: 10.1016/j.jiph.2012.08.001 23164565

[15] Papang R, John AS, Abraham S, Rao PS. A study of steroid-induced diabetes mellitus in leprosy. Indian J Lepr. 2009;81:125–129. 20509340

