# Supplementary material for: Is there a lack of a glucose monitoring and management protocol for preventing hyperglycemia and glucocorticoid-induced diabetes mellitus in leprosy reactions?
Source: PLoS Negl Trop Dis. 2024 Jul 25;18(7):e0012298. doi: 10.1371/journal.pntd.0012298 (PMC11271857; doi:10.1371/journal.pntd.0012298)
Supplement: S1 Table — Abbreviations/notes: BT—Borderline Tuberculoid; BB—Borderline Borderline; BL—Borderline Lepromatous; LL—Lepromatous Leprosy; MB: Multibacillary; PB: Paucibacillary. *—BT classified as MB based on ELISA anti-PGL-1, neural thickening, qPCR slit skin smear, and biopsy. (DOCX) [file pntd.0012298.s001.docx]

| **Group** | | **1^st^ Group** | | **2^nd^ Group** | | **3^rd^ Group** | | **4^th^ Group** | | **Total** | |
| --- | --- | --- | --- | --- | --- | --- | --- | --- | --- | --- | --- |
| ***Cumulative Prednisone usage*** | | ***Up to 4 months*** | | ***> 4 to 8 months*** | | ***> 8 to 12 months*** | | ***> 12 months*** | |  |  |
|  |  | **n** | **%** | **n** | **%** | **n** | **%** | **n** | **%** | **n** | % |
| **Clinical Form** | BT* | 13 | 41.9 | 3 | 13.6 | 1 | 11.1 | 1 | 7.7 | 18 | 24 |
|  | BB | 4 | 12.9 | 5 | 22.7 | 2 | 22.2 | 0 | 0 | 11 | 14.7 |
|  | BL | 4 | 12.9 | 5 | 22.7 | 3 | 33.3 | 6 | 46.2 | 18 | 24 |
|  | LL | 10 | 32.3 | 9 | 40.9 | 3 | 33.3 | 6 | 46.2 | 28 | 37.3 |
|  | **Total** | **31** |  | **22** |  | **9** |  | **13** |  | **75** |  |
| **Type of reaction** | Type 1 | 21 | 67.7 | 13 | 29 | 6 | 66.7 | 7 | 53.8 | 47 | 62.7 |
|  | Type 2 | 10 | 32.3 | 9 | 20 | 3 | 33.3 | 6 | 46.2 | 28 | 37.3 |
|  |  |  |  |  |  |  |  |  |  |  |  |
| **Operational classification** | PB | 0 | 0 | 0 | 0 | 0 | 0 | 0 | 0 | 0 | 0 |
|  | MB | 31 | 100 | 22 | 100 | 9 | 100 | 13 | 100 | 75 | 100 |
|  |  |  |  |  |  |  |  |  |  |  |  |
| **Sex** | Women | 13 | 41.9 | 9 | 40.9 | 4 | 44.4 | 3 | 23.1 | 29 | 38.7 |
|  | Men | 18 | 58.1 | 13 | 59.1 | 5 | 55.6 | 10 | 76.9 | 46 | 61.3 |
|  |  |  |  |  |  |  |  |  |  |  |  |
| **Age** | *Minimum* | 18 | | 18 | | 31 | | 36 | |  |  |
|  | *Median* | 44 | | 46.5 | | 68 | | 52 | |  |  |
|  | *Maximum* | 79 | | 75 | | 84 | | 83 | |  |  |
